# Radiomics‐based ultrasOund Model for differentiating Uterine Sarcomas from leiomyomas (ROMUS): a retrospective pilot Multicenter Italian Trials in Ovarian Cancer (MITO) study

**DOI:** 10.1002/uog.70187

**Published:** 2026-03-06

**Authors:** F. Ciccarone, A. Rizzi, A. Biscione, G. Baldassari, E. H. Tran, T. Pasciuto, F. Moro, G. Zinicola, F. Buonomo, M. Colombin, F. Ghezzi, J. Casarin, R. Mancari, F. Borella, A. Kardhashi, M. Roccio, L. Savelli, R. Cioffi, F. Fanfani, G. Ferrandina, G. Scambia, L. Valentin, D. Lorusso, A. C. Testa

**Affiliations:** ^1^ Gynecologic Oncology Unit, Department of Woman and Child Health and Public Health Fondazione Policlinico Universitario ‘A. Gemelli’ IRCCS Rome Italy; ^2^ Ovarian Cancer Center Candiolo Cancer Institute, FPO‐IRCCS Turin Italy; ^3^ Radiomics G‐STeP Research Core Facility, Fondazione Policlinico Universitario ‘A. Gemelli’ IRCCS Rome Italy; ^4^ Data Collection G‐STeP Research Core Facility, Fondazione Policlinico Universitario ‘A. Gemelli’ IRCCS Rome Italy; ^5^ Section of Hygiene, University Department of Life Sciences and Public Health, Università Cattolica Del Sacro Cuore Rome Italy; ^6^ UniCamillus‐Saint Camillus International University of Health and Medical Sciences Rome Italy; ^7^ Institute for Maternal and Child Health, IRCCS ‘Burlo Garofolo’ Trieste Italy; ^8^ Department of Obstetrics and Gynecology, ‘Filippo Del Ponte’ Hospital University of Insubria Varese Italy; ^9^ Gynecologic Oncology Unit IRCCS Regina Elena National Cancer Institute Rome Italy; ^10^ Obstetrics and Gynecology Unit 1, Department of Surgical Sciences, Sant' Anna Hospital University of Torino Turin Italy; ^11^ Gynecologic Oncology Unit IRCCS Istituto Tumori ‘Giovanni Paolo II’ Bari Italy; ^12^ Department of Obstetrics and Gynecology Fondazione IRCCS Policlinico San Matteo Pavia Italy; ^13^ Department of Obstetrics and Gynaecology Ospedale Morgagni‐Pierantoni Forlì Italy; ^14^ Department of Medical and Surgical Sciences University of Bologna Bologna Italy; ^15^ Department of Gynecological Oncology IRCCS San Raffaele Hospital Milan Italy; ^16^ Section of Obstetrics and Gynecology, University Department of Life Sciences and Public Health, Università Cattolica del Sacro Cuore Rome Italy; ^17^ Department of Obstetrics and Gynecology Skåne University Hospital Malmö Sweden; ^18^ Department of Clinical Sciences Malmö Lund University Malmö Sweden; ^19^ Gynecologic Oncology Unit, Humanitas San Pio X Milan Italy; ^20^ Department of Biomedical Sciences Humanitas University, Pieve Emanuele Milan Italy

**Keywords:** artificial intelligence, leiomyoma, radiomics, sarcomas, ultrasonography, uterine neoplasms

## Abstract

**Objective:**

To develop machine‐learning models that incorporate clinical information and radiomics features extracted from ultrasound images to distinguish uterine sarcomas from leiomyomas.

**Methods:**

This retrospective, multicenter, pilot case–control study included 200 patients (100 with a uterine sarcoma and 100 with a usual‐type leiomyoma, i.e. including no benign leiomyoma variants) who underwent preoperative ultrasound examination between January 2010 and June 2022. The patient cohort was split (70:30) into training and validation sets, with the same proportion of leiomyomas and sarcomas in each subset. We extracted radiomics features belonging to different families: intensity‐based statistical features and textural features. The variables used in model building were patient age and the radiomics features that differed statistically significantly between sarcomas and leiomyomas and that were not redundant based on Spearman's correlation coefficient. Logistic regression, random forest, extreme gradient boosting (XGBoost) and support vector machine models were tested in the model development process. We evaluated the performance of the models in differentiating between sarcomas and leiomyomas using the area under the receiver‐operating‐characteristics curve (AUC), accuracy, sensitivity and specificity. We compared these results to those of subjective assessment by the original ultrasound examiner and to those of two independent expert ultrasound examiners who, blinded to clinical history, reviewed the same grayscale ultrasound images as those used for the radiomics analysis.

**Results:**

Sixty‐three radiomics features were extracted. Of these, eight differed statistically significantly between sarcomas and leiomyomas and were not correlated, so were selected for inclusion in model building. In the validation set, the model that performed best in differentiating between sarcomas and leiomyomas was an XGBoost model integrating patient age and radiomics features. In the validation set, this model had an AUC of 0.93, sensitivity of 0.93 and specificity of 0.83, at a risk‐of‐malignancy cut‐off of 47% (the cut‐off that yielded the highest number of correct classifications based on Youden's index in the training set). The corresponding results for the model integrating only the radiomics features were: AUC of 0.87, sensitivity of 0.87 and specificity of 0.83. Subjective assessment by the original ultrasound examiner had a sensitivity of 0.87 and specificity of 1 in the validation set, while retrospective review of grayscale ultrasound images by ultrasound experts had a sensitivity of 0.87 and specificity of 0.80 (same results for both reviewers).

**Conclusion:**

A model including eight radiomics features and patient age demonstrated reasonably good discriminative and classification performance for distinguishing uterine sarcomas from leiomyomas. Its classification ability was similar to that of subjective assessment by the original ultrasound examiner, being more sensitive but less specific. To confirm the role of radiomics for discriminating between uterine sarcomas and leiomyomas, large prospective studies including benign leiomyoma variants are needed. If good performance of radiomics models can be confirmed, integrating automated radiomics analysis into ultrasound machine software may help ultrasound examiners to discriminate between sarcomas and benign leiomyomas. © 2026 The Author(s). *Ultrasound in Obstetrics & Gynecology* published by John Wiley & Sons Ltd on behalf of International Society of Ultrasound in Obstetrics and Gynecology.

## INTRODUCTION

Uterine sarcomas are rare and aggressive tumors that originate from the smooth muscle of the uterus. They account for approximately 1% of female genital tract malignancies^1^, with a worldwide annual incidence of 1.55–1.95 per 100 000 women^2^. Providing an accurate preoperative diagnosis of uterine sarcoma is essential, as minimally invasive surgery may lead to inadequate treatment and increases the risk of intra‐abdominal dissemination. It is estimated that 0.1–0.3% of patients who undergo surgery for a presumed uterine leiomyoma instead have a uterine sarcoma^3^. Morcellation of unsuspected uterine sarcomas raises the risk of recurrence and mortality^4^, ^5^. Since the symptoms of benign and malignant uterine smooth muscle tumors are similar, clinical diagnosis is challenging. Currently, no validated criteria exist to distinguish accurately between them^6^, ^7^, ^8^, ^9^.

Although magnetic resonance imaging offers high accuracy in characterizing uterine lesions, most available data are based on retrospective studies and results have not been externally validated sufficiently^6^, ^10^, ^11^. Ultrasound is the first‐line diagnostic method for evaluating myometrial lesions, due to its widespread availability, cost‐effectiveness and non‐invasiveness. Typical ultrasound features of uterine sarcomas have been identified^12^ and an algorithm combining clinical and ultrasound findings has been developed to stratify patients with myometrial lesions into low, intermediate and high risk of sarcoma with good accuracy^13^. Additionally, radiomics‐based systems have been developed using ultrasound images from 70 patients with uterine mesenchymal lesions (50 benign and 20 malignant), showing sensitivity of 0.76 to 0.80 and specificity of 0.85 to 0.87 in the differential diagnosis of these tumors^14^. Radiomics involves advanced image analysis techniques that extract quantitative features from medical images beyond those detectable by the human eye, offering insights into the underlying tissue characteristics^15^, ^16^, ^17^. Therefore, combining clinical, ultrasound and radiomics features may potentially enhance diagnostic performance compared with traditional ultrasound evaluation alone.

The primary aim of this study was to develop machine‐learning models that incorporate clinical characteristics and radiomics features extracted from ultrasound images to distinguish uterine sarcomas from leiomyomas. A secondary aim was to compare the performance of the radiomics models with subjective assessment of ultrasound images by experienced ultrasound examiners.

## METHODS

### Study design

This was a multicenter, observational, retrospective case–control pilot study, with Fondazione Policlinico Universitario A. Gemelli IRCCS (Scientific Institute for Hospitalization and Care), Rome, Italy as the co‐ordinating center. The plan was to include 100 patients with a histologically confirmed uterine sarcoma and 100 with a usual‐type leiomyoma (i.e. including no histological benign variants of leiomyoma), ensuring that each leiomyoma was matched to a sarcoma examined with ultrasound during the same year, between January 2010 and June 2022. This matching was done to ensure that ultrasound image quality was similar for leiomyomas and sarcomas, with images obtained using ultrasound machines of a similar generation.

All 173 centers within the Multicenter Italian Trials in Ovarian Cancer and Gynecologic Malignancies (MITO) group, with one additional Swedish center, were invited to participate in the study, provided they could supply the required clinical and ultrasound information and submit ultrasound images that met the inclusion criteria. Clinical information, ultrasound information as described in the original ultrasound reports, ultrasound images and histological data were collected at the participating centers and sent to the co‐ordinating center, where all radiomics and data analyses were carried out. The procedures followed were in accordance with the Declaration of Helsinki and with good clinical practice^18^. Approval of the study was obtained from the local research ethics committee of the coordinating center, as well as from the local ethics committees of each contributing center.

Inclusion criteria were: patient age ≥ 18 years; histological diagnosis (hysterectomy, myoma enucleation or biopsy performed during laparoscopy or laparotomy) of uterine sarcoma or usual‐type leiomyoma; preoperative transvaginal, transrectal or transabdominal ultrasound examination performed within 3 months before surgery, between January 2010 and June 2022; and availability of at least one preoperative grayscale ultrasound image in Digital Imaging and Communications in Medicine (DICOM) format. We excluded patients with poor quality images, those with images in which calipers were visible or text covered the tumor and those with only color Doppler images. Patients with a histological diagnosis of benign leiomyoma variants or uterine smooth muscle tumors of uncertain malignant potential (STUMP) were not included.

Clinical information (including stage and histological subtype of the tumor) and ultrasound information were collected at each participating center, sent to the coordinating center in an Excel file (Microsoft Corp., Redmond, WA, USA) and then managed by the coordinating center using Research Electronic Data Capture (REDCap) tools hosted at the Fondazione Policlinico Universitario A. Gemelli, IRCCS (https://redcap‐irccs.policlinicogemelli.it)^19^, ^20^. All data and images were handled in compliance with the general data protection regulation (GDPR). The principal investigator at each center extracted information on the ultrasound features of the lesions from the original ultrasound reports or, if information on some of the ultrasound variables was missing from the ultrasound reports, by reviewing the saved images to provide the missing information. Both the original ultrasound examiners and the principal investigators were European Federation of Societies for Ultrasound in Medicine and Biology (EFSUMB) Level‐II or Level‐III operators^21^.

The ultrasound features were described using the Morphological Uterus Sonographic Assessment (MUSA) terminology^22^. The following prespecified ultrasound information was recorded: single myometrial lesion (yes/no); visible normal myometrium (yes/no); maximum tumor diameter (in mm); lesion borders (regular/irregular/impossible to say); echostructure (uniform/non‐uniform); echogenicity (hypoechogenic/isoechogenic/hyperechogenic/mixed); color score (1/2/3/4); presence of cystic areas within the lesion (yes/no); echogenicity of cystic content if present (anechoic/low level/ground glass/hemorrhagic/more than one type of echogenicity); regularity of the walls of cystic areas (regular/irregular); presence of acoustic shadows (yes/no); presence of calcifications (yes/no); and presence of ascites (yes/no). The presence of cooked appearance (yes/no), which refers to an ultrasound feature suggestive of structural deterioration following tissue necrosis, was also recorded^12^.

The results of subjective assessment (pattern recognition) of the original ultrasound examiner at the time of the scan, as documented in the original ultrasound report, was recorded and categorized as malignant, indeterminate (uncertain) or benign. The same grayscale images as those used for radiomics analysis were also reviewed by two independent gynecologists (G.Z., L.V.), highly experienced in gynecological ultrasound (EFSUMB^21^ Level II and III, respectively). They classified the myometrial lesion as malignant, indeterminate (uncertain) or benign without access to any information other than the single ultrasound image per tumor. This ensured fair comparison with the radiomics analysis, which was based solely on one grayscale ultrasound image per tumor.

The stage of the uterine sarcomas was determined at the local centers and described according to the International Federation of Obstetrics and Gynecology (FIGO) staging system^23^. Histological subtypes were reported using the World Health Organization (WHO) 2020 classification^24^. The histological diagnoses were provided by pathologists in the local centers. All centers had pathologists specifically dedicated to gynecology. No central pathology review was performed.

### Image segmentation and preprocessing

**Figure 1:**
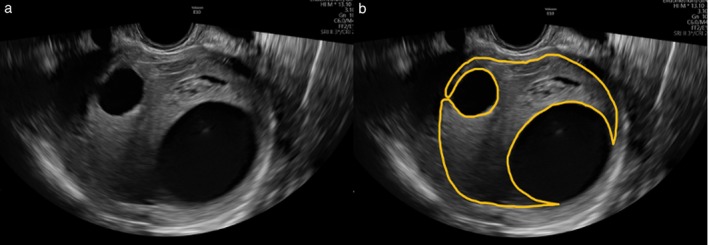
Grayscale ultrasound images of sarcoma without (a) and with (b) manually segmented tumor mask (yellow contour) obtained using Aliza Medical version 2.3.10 software. Only solid tissue was included in region of interest; cystic areas larger than 5 mm in maximum diameter were excluded.

Transabdominal, transvaginal or transrectal grayscale ultrasound images of the myometrial lesion in DICOM format were accepted for analysis. If there were multiple images of the same tumor, one of the authors at the coordinating center (F.C.) selected the highest‐quality image for radiomics analysis, based on criteria such as the clearest delineation of tumor borders, largest section of the tumor, optimal image resolution and clarity of internal structures. The region of interest (ROI) in each image was segmented manually by one of two EFSUMB^21^ Level‐II examiners (A.R., A.B.), using Aliza software, version 2.3.10 (Aliza Medical Imaging, Bonn, Germany). All ROIs were then reviewed by an EFSUMB Level‐III examiner (F.C.) and corrected if necessary. The ROI included only the solid component of the mass; cysts larger than 5 mm in maximum diameter were excluded from the ROI (Figure 1), in order to focus specifically on the ultrasound characteristics of the solid tissue.

All ultrasound images were then preprocessed to reduce speckle noise using a Wiener filter with kernel size 3 × 3^25^. Image intensities were standardized for each subject using *Z*‐score normalization, considering the mean and SD of the intensities within the ROI.

### Extraction of radiomics features

Extraction of radiomics features was performed using RStudio (R version 4.2.2)^26^, ^27^ as the coding environment. Radiomics features were extracted from ROIs in the preprocessed images using MODDICOM^28^, an open‐source R library developed by the Radiomics Research Core facility of the Fondazione Policlinico Universitario ‘A. Gemelli’ IRCCS, Rome, Italy, and validated within the Image Biomarker Standardization Initiative^29^, to ensure reproducibility and methodological robustness of the radiomics features extraction pipeline. The extracted features belonged to two different families: first‐order intensity‐based statistical features (F_stat) and textural features. First‐order statistical features described the statistical properties of the image gray‐level histogram within the ROI. Textural features described the properties of the local spatial distribution of gray levels inside the ROI. The textural features were computed based on the following matrices: (1) gray‐level size zone matrix (‘F_szm’), which represents the number of groups (zones) of neighboring pixels with the same gray level; (2) gray‐level run‐length matrix (‘F_rlm’), which represents the length of a consecutive sequence of pixels with the same gray level; and (3) gray‐level co‐occurrence matrix (‘F_cm’), in which the co‐occurrence of gray levels within neighboring pixels is reported.

### Statistical analysis

We report categorical data as frequencies and percentages and summarize quantitative data using mean and SD. We tested the statistical significance of differences in clinical and ultrasound characteristics between sarcomas and leiomyomas using the chi‐square test or Fisher's exact test for categorical data and Student's *t*‐test for continuous data. Stata software version 17.0 (STATA/BE 17.0 for Windows, StataCorp. LLC, College Station, TX, USA) was used for these statistical analyses. *P* < 0.05 was considered statistically significant.

The cohort of patients was split randomly into training and hold‐out validation sets with a ratio of 70:30, and with the same proportion of leiomyomas (benign lesions) and sarcomas (malignant lesions) within each subset: 140 patients (70 sarcomas and 70 leiomyomas) in the training set and 60 patients (30 sarcomas and 30 leiomyomas) in the validation set. Radiomics feature selection was performed on the training set via univariate analysis with the Wilcoxon–Mann–Whitney statistical test. *P*‐values were corrected using Benjamini–Hochberg correction for multiple comparisons (significance level, 0.05). The correlation between radiomics features that exhibited statistically significant differences between sarcomas and leiomyomas was investigated using the Spearman correlation coefficient. To mitigate multicollinearity, when the correlation between two features exceeded 0.6, only one was retained, the feature with the lowest mean correlation with all other features in the correlation matrix. This selection process was iterated until all pairwise correlations were below the 0.6 threshold, resulting in a final set of non‐redundant, uncorrelated features.

### Radiomics modeling and model validation

Preprocessing, feature selection, radiomics modeling and model validation were performed using Python version 3.10.14^30^ in Visual Studio Code as the coding environment^31^. To bring the values of the selected radiomics features to a common scale, for each patient, each feature value was normalized using the mean and SD of that feature across all patients (*Z*‐score normalization). To discriminate between sarcomas and leiomyomas, two families of models were developed: radiomics models, which included as input variables only the radiomics features selected in the training set, and clinical–radiomics models, which included the selected radiomics features plus the patient's age. Each set of selected radiomics features, with and without patient age, was used to train four different machine‐learning classifiers: logistic regression, random forest, support vector machine (SVM) and extreme gradient boosting (XGBoost). To identify the optimal set of model hyperparameters (i.e. parameters characteristic of the machine‐learning model, which modify and guide the learning process), fine tuning was performed with a randomized grid search using a 5‐fold crossvalidation over the training set.

All models were tested on the validation set. Their ability to discriminate between sarcomas and leiomyomas was described using the area under the receiver‐operating‐characteristics curve (AUC). We also calculated classification metrics (accuracy, sensitivity and specificity) using the best risk‐of‐malignancy cut‐off, i.e. the cut‐off that provided the largest number of correct classifications according to Youden's index^32^. We used bootstrapping to estimate the 95% CIs of the AUCs and normal approximation to estimate the 95% CIs for the classification metrics. For both the radiomics and the clinical–radiomics models, the optimal machine‐learning model was chosen based on the AUCs in the training set. If more than one machine‐learning model exhibited the same AUC value, the machine‐learning model that yielded the highest sensitivity was selected. When calculating the classification performance of subjective assessment, uncertain diagnoses were classified as malignant. Cohen's kappa^33^ was calculated on the validation set to estimate the agreement between the radiomics model and subjective assessment by each of the two expert ultrasound examiners who reviewed the same images as those used to validate the radiomics model.

We evaluated the calibration of the models by calculating the calibration intercept and slope, which were then used to generate calibration curves in the validation set. The calibration intercept indicates whether risks are generally overestimated (intercept < 0) or underestimated (intercept > 0). The calibration slope reveals whether predicted risks are too extreme, i.e. low risks are underestimated and high risks are overestimated (slope < 1), or too moderate, i.e. low risks are overestimated and high risks are underestimated (slope > 1)^34^. Calibration analysis was performed using RStudio (Posit PBC, Boston, MA, USA) with R version 4.2.2 (R Foundation for Statistical Computing, Vienna, Austria)^26^, ^27^.

### Difference in radiomics features between normal myometrium and tumor

A subanalysis was conducted to develop a delta‐feature radiomics model for discrimination between sarcomas and leiomyomas. This model used delta‐radiomics features, i.e. features reflecting differences in radiomics features between the tumor and the surrounding tumor‐free myometrium. For this subanalysis, only patients whose images showed clear evidence of surrounding tumor‐free myometrium were included. For each image, a ROI corresponding to the visible tumor‐free myometrium was delineated. Features from the ROI of the tumor‐free myometrium were extracted and the delta‐radiomics features were calculated by dividing the value for each radiomics feature of the myometrial mass by that of the tumor‐free myometrium. Radiomics feature selection was performed based on univariate analysis using the Wilcoxon–Mann–Whitney statistical test followed by the Boruta algorithm. The Boruta algorithm is a robust and comprehensive method, based on a machine‐learning algorithm, that relies on feature importance scores. It was used to identify all relevant delta‐features^35^. To mitigate multicollinearity among the delta‐features, the same procedure using the Spearman correlation coefficient as that used for development of the radiomics models was applied. The selected delta‐features were used as variables in a stepwise logistic regression analysis based on the Akaike information criteria (AIC) to create a model including only delta‐radiomics features^36^. The model was fitted on this subset of the dataset and then internally validated using 3‐fold crossvalidation. The ability of the model to discriminate between sarcoma and leiomyoma was quantified by calculating the AUC. In addition, the accuracy, sensitivity and specificity of the model when using the risk‐of‐malignancy cut‐off that maximized the number of correct classifications (Youden's cut‐off) were computed. For the crossvalidation, the metrics were computed as the mean and SD across all folds. The 95% CIs for AUCs were calculated using bootstrapping, while the 95% CIs for accuracy, sensitivity and specificity were estimated using a normal approximation. For the model fitting, the 95% CIs were calculated. All analyses regarding delta‐radiomics were performed in RStudio (R version 4.2.2)^26^, ^27^.

## RESULTS

Twenty‐nine of the 174 invited centers responded to our invitation to participate in this study. However, only 13 of these centers were able to provide clinical information and ultrasound images that met the stringent inclusion criteria for radiomics analysis. These 13 centers together provided data on 100 patients with uterine sarcoma and 100 with usual‐type leiomyoma (Table S1). All patients had undergone transvaginal or transrectal ultrasound examination supplemented with a transabdominal scan, if needed, and all ultrasound examinations had been carried out by an EFSUMB Level‐II or Level‐III examiner^21^ using high‐end ultrasound equipment: Samsung HS70A, Samsung‐elite, Samsung HERA I10 or Samsung WS80A (Samsung Medison Co. Ltd., Seoul, South Korea); Voluson E10, Voluson E8, Voluson E6 or Voluson 730 (GE Healthcare, Zipf, Austria); Hitachi arietta S70 (Hitachi, Tokyo, Japan); Canon Aplio i700 (Canon Medical Systems Europe BV, Zoetermeer, The Netherlands); or Esaote XP 8 (Esaote, Genova, Italy). The frequency of the transvaginal probes varied between 5.0 and 9.0 MHz and that of the transabdominal probes varied between 3.5 and 5.0 MHz.

The clinical characteristics of the study population are summarized in Table 1. The mean ± SD age at diagnosis for the entire study population was 52 ± 13 years. Patients with sarcomas were older than those with leiomyomas (59 ± 13 *vs* 45 ± 9 years; *P* < 0.0001). Most (68%) patients with sarcomas were postmenopausal, whereas patients with leiomyomas were predominantly premenopausal (89%). Patients with sarcomas were more frequently symptomatic compared to patients with leiomyomas. The most common symptoms were abnormal uterine bleeding (56% in case of sarcoma *vs* 36% in case of leiomyoma; *P* = 0.007) and abdominal or pelvic pain (44% *vs* 18%; *P* = 0.0001).

**Table 1:** Clinical characteristics of study population of 200 patients with uterine sarcoma or usual‐type leiomyoma

Characteristic	All tumors (*n* = 200)	Leiomyoma (*n* = 100)	Sarcoma (*n* = 100)	*P*
Age at diagnosis (years)	52 ± 13	45 ± 9	59 ± 13	< 0.0001
BMI (kg/m^2^)*	25.2 ± 4.9	24.4 ± 3.4	25.9 ± 6.1	0.05
Postmenopausal	79 (39.5)	11 (11.0)	68 (68.0)	< 0.0001
Parous	117/198 (59.1)	41/100 (41.0)	76/98 (77.6)	< 0.0001
Symptoms†				
Asymptomatic	35 (17.5)	25 (25.0)	10 (10.0)	0.009
Abnormal uterine bleeding	92 (46.0)	36 (36.0)	56 (56.0)	0.007
Abdominal or pelvic pain	62 (31.0)	18 (18.0)	44 (44.0)	0.0001
Abdominal swelling or pressure	54 (27.0)	26 (26.0)	28 (28.0)	0.752
Other	7 (3.5)	4 (4.0)	3 (3.0)	1
Fertility history				
Infertility (> 12 months)	4 (2.0)	4 (4.0)	0 (0)	0.120
Two or more miscarriages	4 (2.0)	2 (2.0)	2 (2.0)	1
Type of surgery				
Hysterectomy	156 (78.0)	67 (67.0)	89 (89.0)	< 0.0001
Myomectomy	35 (17.5)	33 (33.0)	2 (2.0)	< 0.0001
Biopsy performed during laparoscopy or laparotomy	9 (4.5)	0 (0)	9 (9.0)	0.003
Histotype				—
Leiomyoma	100 (50.0)	100 (100)	0 (0)	
Leiomyosarcoma	58 (29.0)	0 (0)	58 (58.0)	
Endometrial stromal sarcoma	32 (16.0)	0 (0)	32 (32.0)	
Undifferentiated sarcoma	8 (4.0)	0 (0)	8 (8.0)	
Müllerian adenosarcoma	2 (1.0)	0 (0)	2 (2.0)	
FIGO stage				—
I	—	NA	65 (65.0)	
II	—	NA	10 (10.0)	
III	—	NA	8 (8.0)	
IV	—	NA	17 (17.0)	

Data are presented as mean ± SD, *n* (%) or *n*/*N* (%). *P*‐values were calculated using two‐sided chi‐square test, Fisher's exact test or Student's *t*‐test, as appropriate.

* Information available for 162/200 patients, including 83 with leiomyoma and 79 with sarcoma.

† In some cases, there was more than one symptom. BMI, body mass index; FIGO, International Federation of Obstetrics and Gynaecology^23^; NA, not applicable.

**Figure 2:**
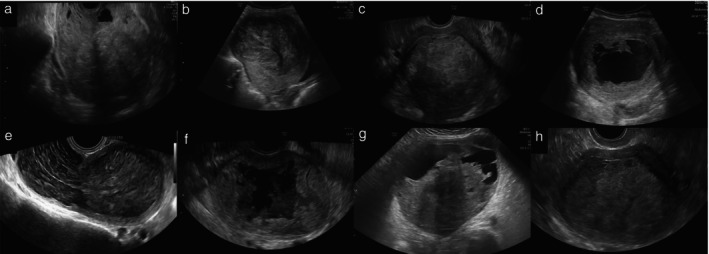
Grayscale ultrasound images of histologically confirmed uterine sarcomas. On histology, four were identified as leiomyosarcomas (a–d), two as undifferentiated uterine sarcomas (e,f) and two as endometrial stromal sarcomas (g,h). Sarcomas typically present as large myometrial masses with non‐uniform, heterogeneous echogenicity (a–h), internal cystic areas (a,d,f–h) and no acoustic shadows (b–f,h).

**Figure 3:**
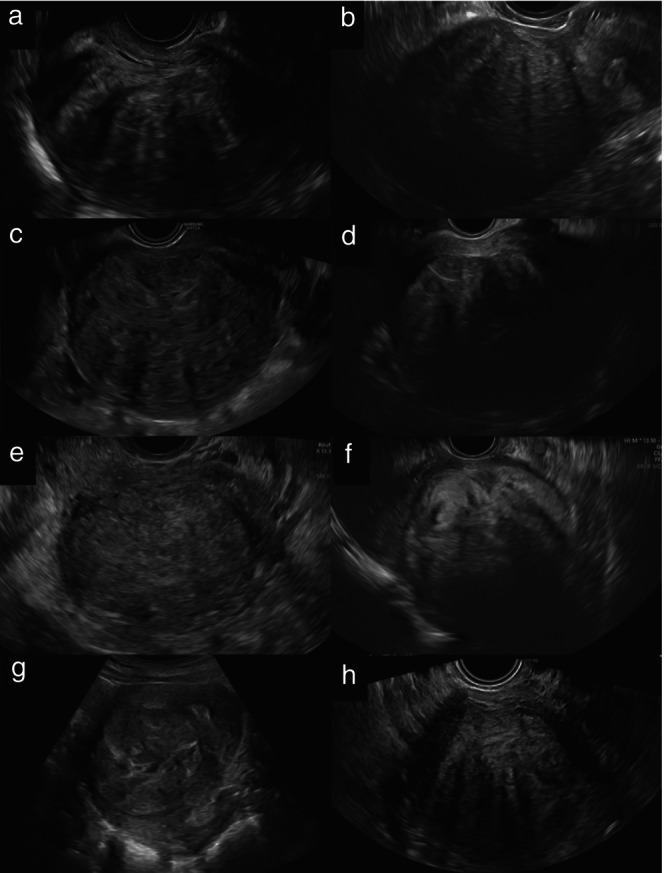
Grayscale ultrasound images of histologically confirmed usual‐type uterine leiomyomas. Leiomyomas are typically solid lesions with heterogeneous echogenicity (a–d,f–h) and acoustic shadows (a–d,f,h). Some leiomyomas may exhibit cystic areas (g) or homogeneous echogenicity (e), which can complicate differential diagnosis.

The ultrasound characteristics of the tumors as described in the original ultrasound reports (or, if information on some variables was missing from the original ultrasound report, as observed on review of saved ultrasound images by the principal investigator at each center) are summarized in Table 2. Examples of ultrasound images of uterine sarcomas from our series are shown in Figure 2 and images of benign usual‐type uterine leiomyomas (not benign variants) are shown in Figure 3. Among the 100 sarcomas, there were 58 leiomyosarcomas, 32 endometrial stromal sarcomas, eight undifferentiated sarcomas and two Müllerian adenosarcomas. Sarcomas appeared as solitary lesions more often than did leiomyomas (81% *vs* 46%; *P* < 0.0001). They were larger than leiomyomas (mean ± SD largest tumor diameter, 112 ± 59 *vs* 73 ± 38 mm; *P* < 0.0001) and they were more often moderately or richly vascularized (color score 3 or 4, 70% *vs* 39%; *P* < 0.0001). Irregular borders were reported in 66% of sarcomas *vs* 1% of leiomyomas (*P* < 0.0001). Sarcomas exhibited non‐uniform echogenicity in almost all (95%) cases and contained cystic areas in 64% of cases, while leiomyomas exhibited non‐uniform echogenicity in 59% of cases, with cystic areas in 11%. Sarcomas manifested acoustic shadows less frequently than did leiomyomas (21% *vs* 83%; *P* < 0.0001). The cooked appearance was observed in 48% of sarcomas *vs* 2% of leiomyomas (*P* < 0.0001). The original ultrasound examiner correctly classified 80 of 100 sarcomas as malignant (considering as malignant any cases that they classified as uncertain), corresponding to a sensitivity of 0.80 (95% CI, 0.72–0.88) and 95 of 100 leiomyomas as benign, corresponding to a specificity of 0.95 (95% CI, 0.91–0.99) (Table 3). Retrospective review using subjective assessment of the same grayscale ultrasound images as those used for radiomics analysis had a sensitivity of 0.87 (95% CI, 0.80–0.94) and specificity of 0.83 (95% CI, 0.76–0.90) for Reviewer 1 and a sensitivity of 0.89 (95% CI, 0.83–0.95) and specificity of 0.77 (95% CI, 0.69–0.85) for Reviewer 2.

**Table 2:** Ultrasound characteristics of uterine sarcomas and usual‐type leiomyomas as described in original ultrasound reports, or according to review of saved ultrasound images by principal investigator at each center*

Characteristic	All tumors (*n* = 200)	Leiomyoma (*n* = 100)	Sarcoma (*n* = 100)	*P*
Single lesion	118/188 (62.8)	45/98 (45.9)	73/90 (81.1)	< 0.0001
Visible normal myometrium	170/198 (85.9)	96/98 (98.0)	74/100 (74.0)	< 0.0001
Maximum tumor diameter (mm)	93 ± 54	73 ± 38	112 ± 59	< 0.0001
Tumor border				< 0.0001
Regular	133 (66.5)	99 (99.0)	34 (34.0)	
Irregular	67 (33.5)	1 (1.0)	66 (66.0)	
Echostructure				< 0.0001
Uniform	46 (23.0)	41 (41.0)	5 (5.0)	
Non‐uniform	154 (77.0)	59 (59.0)	95 (95.0)	
Echogenicity				0.305
Hypoechogenic	71 (35.5)	37 (37.0)	34 (34.0)	
Isoechogenic	71 (35.5)	38 (38.0)	33 (33.0)	
Hyperechogenic	15 (7.5)	4 (4.0)	11 (11.0)	
Mixed	43 (21.5)	21 (21.0)	22 (22.0)	
Acoustic shadows present	104 (52.0)	83 (83.0)	21 (21.0)	< 0.0001
Calcifications present	21 (10.5)	13 (13.0)	8 (8.0)	0.356
Cooked appearance	50 (25.0)	2 (2.0)	48 (48.0)	< 0.0001
Presence of cystic areas	75 (37.5)	11 (11.0)	64 (64.0)	< 0.0001
Cystic content				1
Anechoic	62/75 (82.7)	10/11 (90.9)	52/64 (81.3)	
Low‐level	8/75 (10.7)	1/11 (9.1)	7/64 (10.9)	
Ground‐glass	0/75 (0)	0/11 (0)	0/64 (0)	
Hemorrhagic	2/75 (2.7)	0/11 (0)	2/64 (3.1)	
More than one type	3/75 (4.0)	0/11 (0)	3/64 (4.7)	
Walls of cystic areas				0.0005
Regular	25/75 (33.3)	9/11 (81.8)	16/64 (25.0)	
Irregular	50/75 (66.7)	2/11 (18.2)	48/64 (75.0)	
Color score				< 0.0001
1	7/195 (3.6)	6/96 (6.3)	1/99 (1.0)	
2	82/195 (42.1)	53/96 (55.2)	29/99 (29.3)	
3	75/195 (38.5)	32/96 (33.3)	43/99 (43.4)	
4	31/195 (15.9)	5/96 (5.2)	26/99 (26.3)	
Original ultrasound examiner's diagnosis (subjective assessment)				< 0.0001
Benign	115 (57.5)	95 (95.0)	20 (20.0)	
Malignant	75 (37.5)	2 (2.0)	73 (73.0)	
Uncertain	10 (5.0)	3 (3.0)	7 (7.0)	

Data are presented as *n*/*N* (%), mean ± SD or *n* (%). *P*‐values were calculated using chi‐square test, Fisher's exact test or Student's *t*‐test, as appropriate. No patient presented with ascites.

* In case of missing information on some ultrasound variables in ultrasound reports.

**Table 3:** Performance of subjective assessment by original ultrasound (US) examiner and two independent expert US examiners, and of radiomics and clinical–radiomics models, in distinguishing between uterine sarcomas and leiomyomas in training (140 patients) and validation (60 patients) sets

	Subjective assessment									Machine‐learning model: XGBoost			
	Expert US examiner I			Expert US examiner II			Original US examiner			Clinical–radiomics model		Radiomics model	
Parameter	Training	Validation	Total	Training	Validation	Total	Training	Validation	Total	Training	Validation	Training	Validation
AUC	NA	NA	NA	NA	NA	NA	NA	NA	NA	0.98 (0.97–1)	0.93 (0.86–0.99)	0.93 (0.88–0.96)	0.87 (0.76–0.96)
Accuracy	0.86 (0.80–0.92)	0.83 (0.74–0.93)	0.85 (0.80–0.90)	0.83 (0.77–0.89)	0.83 (0.74–0.93)	0.83 (0.78–0.88)	0.85 (0.79–0.90)	0.93 (0.87–1)	0.87 (0.83–0.92)	0.94 (0.90–0.98)	0.88 (0.70–0.97)	0.86 (0.81–0.92)	0.85 (0.76–0.94)
Sensitivity	0.87 (0.79–0.95)	0.87 (0.75–0.99)	0.87 (0.80–0.94)	0.90 (0.83–0.97)	0.87 (0.75–0.99)	0.89 (0.83–0.95)	0.77 (0.67–0.87)	0.87 (0.75–0.99)	0.80 (0.72–0.88)	0.90 (0.83–0.97)	0.93 (0.84–1)	0.89 (0.81–0.96)	0.87 (0.74–0.99)
Specificity	0.84 (0.76–0.93)	0.80 (0.66–0.94)	0.83 (0.76–0.90)	0.76 (0.66–0.86)	0.80 (0.66–0.94)	0.77 (0.69–0.85)	0.92 (0.87–0.99)	1 (1–1)	0.95 (0.91–0.99)	0.97 (0.93–1)	0.83 (0.70–0.97)	0.84 (0.76–0.93)	0.83 (0.70–0.97)

Numbers in parentheses are 95% CI. For the models, accuracy, sensitivity and specificity were calculated for a 47% risk of malignancy, i.e. best cut‐off based on Youden's index obtained in training set and applied in validation set. For subjective assessment, when calculating accuracy, sensitivity and specificity, uncertain diagnoses were classified as malignant. AUC, area under receiver‐operating‐characteristics curve; NA, not applicable; XGBoost, extreme gradient boosting.

### Radiomics features extraction and selection

In total, 200 preoperative ultrasound images from 200 tumors were analyzed, including 42 transabdominal and 158 transvaginal images. Seventy‐four radiomics features were extracted for each image, including 17 statistical and 57 textural features. Features that contained no usable information, either because they were missing for all patients (not a number) or because they had the same value (zero) for all patients, were excluded from the analysis as they did not provide meaningful discriminative information, leaving 63 radiomics features that were included in the analysis. In the training set, eight radiomics features were selected (Appendix S1) which differed statistically significantly between sarcomas and leiomyomas and were not correlated (Wilcoxon–Mann–Whitney and Benjamini–Hochberg tests and Spearman correlation analysis): ‘F_stat.rms’ (*P* = 0.000010), ‘F_cm.joint.entr’ (*P* = 0.017299), ‘F_cm.inv.var’ (*P* = 0.000157), ‘F_cm.clust.shade’ (*P* = 0.000010), ‘F_cm.info.corr.2’ (*P* = 0.0 01367), ‘F_rlm.lrhge’ (*P* = 0.001107), ‘F_szm.lze’ (*P* = 0.001394) and ‘F_szm.glnu’ (*P* = 0.001540). These eight radiomics features were included in the model building, together with patient age.

### Radiomics modeling and validation of diagnostic performance

**Figure 4:**
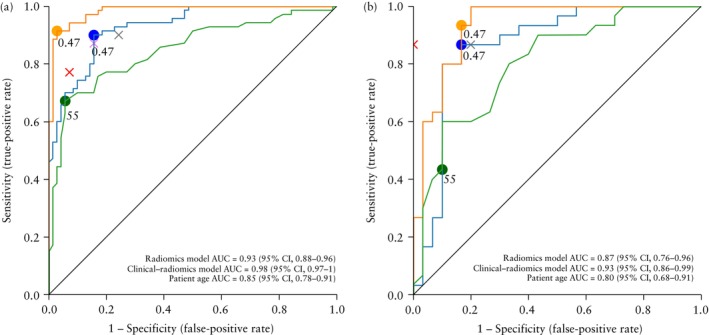
Receiver‐operating‐characteristics (ROC) curves showing performance in differentiating uterine sarcomas from leiomyomas of radiomics model (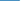), clinical–radiomics model (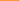) and patient age (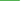) in: (a) training (*n* = 140) and (b) validation (*n* = 60) sets. Best cut‐offs according to Youden's index, calculated in training set and applied in validation set, are indicated for each plot (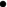). For age, cut‐off indicates a threshold of 55 years; 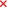 indicates classification performance of original ultrasound examiner using subjective assessment, 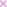 and 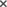 indicate classification performance of independent expert reviewers I and II, respectively. Reviewers had the same information as the radiomics analysis, i.e. only one grayscale image per tumor and no other information, to enable relevant comparison with radiomics‐only model. In the validation set (b), the two reviewers had the same sensitivity and specificity.

The machine‐learning model that performed best for both the radiomics and the clinical–radiomics model types was XGBoost. The discrimination and classification performance for the XGBoost radiomics and clinical–radiomics models and for subjective assessment in both the training and validation sets are presented in Table 3. In both the training and validation sets, the AUC for the clinical–radiomics model was greater than that for the radiomics model (Table 3, Figure 4). We also evaluated the discriminative ability of the variable patient age alone, which resulted in an AUC of 0.85 (95% CI, 0.78–0.91) in the training set and 0.80 (95% CI, 0.68–0.91) in the validation set, lower than that of the radiomics and clinical–radiomics models. The best risk‐of‐malignancy cut‐off (based on Youden's index) in the training set was 47% for both models. Applying a 47% risk‐of‐malignancy cut‐off in the validation set, the XGBoost clinical–radiomics model showed better performance in classifying a lesion as sarcoma (malignant lesion) or leiomyoma (benign lesion), than did the radiomics model, with a sensitivity of 0.93 (95% CI, 0.84–1), specificity of 0.83 (95% CI, 0.70–0.97) and accuracy of 0.88 (95% CI, 0.70–0.97) *vs* a sensitivity of 0.87 (95% CI, 0.74–0.99), specificity of 0.83 (95% CI, 0.70–0.97) and accuracy of 0.85 (95% CI, 0.76–0.94). The specificity at a sensitivity of 1.0 was 0.80 for the clinical–radiomics model *vs* 0.43 for the radiomics model in the validation set. On subjective assessment at ultrasound, the original ultrasound examiner had a sensitivity of 0.87 (95% CI, 0.75–0.99), specificity of 1 (95% CI, 1–1) and accuracy of 0.93 (95% CI, 0.87–1) in classifying a lesion as sarcoma (malignant lesion) or leiomyoma (benign lesion), in the validation set. On retrospective review using pattern recognition (subjective assessment) of the same grayscale ultrasound images as those used for radiomics analysis, the reviewers performed similarly, each having a sensitivity of 0.87 (95% CI, 0.75–0.99), specificity of 0.80 (95% CI, 0.66–0.94) and accuracy of 0.83 (95% CI, 0.74–0.93) in classifying a lesion as sarcoma (malignant lesion) or leiomyoma (benign lesion), in the validation set. The agreement (Cohen's kappa) between the classification by the radiomics model and the classification by each of the expert ultrasound examiners in the validation set was 0.70 (same kappa value for both experts).

**Figure 5:**
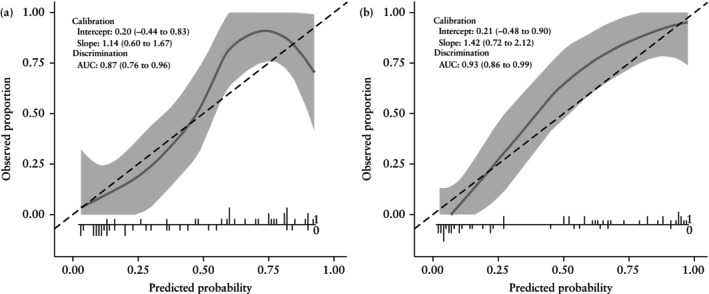
Calibration curves for radiomics (a) and clinical–radiomics (b) models in validation set. Calibration intercept indicates whether risks are generally overestimated (intercept < 0) or underestimated (intercept > 0). Calibration slope reveals whether predicted risks are overly extreme (slope < 1) or too moderate (slope > 1). Shaded area is 95% CI around the estimated calibration curve. 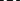, ideal calibration; 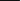, flexible calibration (Loess). Discriminative ability of the models, expressed in terms of area under the receiver‐operating‐characteristics curve (AUC), is also reported. Histogram at the bottom shows how predicted probabilities are distributed across patients, with each vertical line representing an individual case: lines extending below horizontal axis correspond to patients with benign outcome (0), while lines extending above it correspond to patients with malignant outcome (1). Areas where vertical lines are densely packed indicate probability ranges in which many predictions are concentrated.

Looking at the calibration curves (Figure 5), both the clinical–radiomics model and the radiomics model underestimated the risk of malignancy (intercept > 0) and the predicted risks were too moderate (suggested by slope > 1) in the validation set. However, the 95% CIs around the calibration curves were wide (Figure 5), indicating considerable uncertainty in the estimated model calibration.

The discriminative and classification performance of the machine‐learning classifiers SVM, random forest and logistic regression in the training and validation sets are given in Tables S2 and S3 and Figures S1 and S2.

The subanalysis of delta‐radiomics features included 32 patients with leiomyomas and 32 patients with sarcomas who had adequate ultrasound images of tumor‐free myometrium. Two textural features were selected for inclusion in a delta‐feature radiomics model: F_cm.energy (*P* = 0.04) and F_rlm.glnu (*P* = 0.0002) (Figure S3). The best risk‐of‐malignancy cut‐off for the delta‐radiomics model was 0.49. The results of the internal 3‐fold crossvalidation showed the delta‐feature radiomics model to have a mean ± SD AUC of 0.78 ± 0.07, sensitivity of 0.71 ± 0.01, specificity of 0.74 ± 0.08 and accuracy of 0.73 ± 0.09 (Table S4).

## DISCUSSION

We have shown that sarcomas and leiomyomas exhibit significantly different radiomics features extracted from ultrasound images. The clinical–radiomics model, which incorporated eight radiomics features and patient age, demonstrated reasonably good discrimination and classification performance in the validation set. Its classification ability was similar to that of subjective assessment by the original ultrasound examiner (being more sensitive, but less specific). The radiomics model had sensitivity and specificity similar to those of two expert reviewers who, without clinical information, analyzed the same grayscale ultrasound images as those used for the radiomics analysis. Our results do not support that comparing radiomics features of the tumor with those of the surrounding normal myometrium in the same patient would be useful for discriminating between malignant sarcomas and benign leiomyomas.

To the best of our knowledge, this is the largest study to date evaluating radiomics analysis of ultrasound images of uterine myometrial lesions. However, this study has limitations. In this retrospective pilot case–control study, 50% of the tumors were sarcomas, while, in reality, sarcomas account for less than 1% of all myometrial tumors^1^. Moreover, excluding benign leiomyoma variants from the control group and STUMP from the malignant group simplified the discrimination between benign and malignant tumors. The decision to include only usual‐type leiomyomas and sarcomas was due to the exploratory nature of this study, which was designed in 2020, when data on radiomics applied to ultrasound images were still very limited. Our goal was to investigate whether there were any detectable differences between uterine sarcomas and common uterine leiomyomas. This design limits the applicability of our model in the clinical setting. The use of only high‐quality DICOM images ensured dataset uniformity, but limits the generalizability of the results to the clinical setting, in which image quality might be suboptimal. We made the decision to include only one ultrasound image per tumor, despite the fact that several studies on the use of artificial intelligence to discriminate between benign and malignant gynecological tumors have included multiple images per tumor^16^. This decision was made because using multiple images may introduce bias, due to the possibility that unusual ultrasound patterns might be over‐represented in cases with more than one image. Moreover, for some tumors in this study, only a single image fulfilling the inclusion criteria was available.

The clinical and ultrasound characteristics of patients with sarcomas in this study were consistent with those reported in previous studies, in that the women with sarcomas were older and more often postmenopausal compared to the women with benign leiomyomas^37^, ^38^. In agreement with other studies^12^, sarcomas appeared on ultrasound examination as large myometrial masses with irregular margins, non‐uniform echogenicity and internal cystic areas. A cooked appearance, described previously as an ultrasound feature of sarcomas^12^, ^13^, was identified in a large proportion of our cases, while this was rarely observed in the leiomyomas. This feature has been proposed to indicate necrosis, which may partly explain the differences in radiomics textural features between the two groups.

There is growing interest in the application of radiomics analysis to medical imaging in oncology. In gynecological oncology, research has focused primarily on cervical and ovarian cancers, with recent studies highlighting the potential of radiomics applied to ultrasound images^15^, ^39^, ^40^, ^41^. Most studies, however, have focused on magnetic resonance imaging, computed tomography and positron emission tomography/computed tomography^42^, ^43^, ^44^. Roller *et al*.^45^ developed a machine‐learning model that combined clinical variables with radiomics data from T2‐weighted magnetic resonance images in 108 patients (69 leiomyomas, 39 leiomyosarcomas); it achieved a diagnostic accuracy of over 90% and outperformed subjective assessment by two radiologists. To our knowledge, the study by Chiappa *et al*.^14^ is the only published study in which radiomics analysis has been applied to ultrasound images with the aim of differentiating uterine leiomyomas from sarcomas. Their case–control study included 50 benign leiomyomas and 20 sarcomas. Their best model, which incorporated only radiomics features and no clinical variables, achieved an AUC of 0.86, accuracy of 0.85, sensitivity of 0.80 and specificity of 0.87 (with the cut‐off for classification metrics unspecified). This discriminative and classification performance is similar to that of our radiomics model, but inferior to that of our clinical–radiomics model, which also included patient age. It is noteworthy that our radiomics model at its best cut‐off had similar sensitivity and only slightly better specificity compared with two expert reviewers who analyzed the same grayscale ultrasound images (one per tumor) as those used for radiomics analysis, without access to any additional information. The classification performance of the original ultrasound examiners was superior to that of the two reviewers of the ultrasound images, underscoring the importance of performing live scans and obtaining multiple images of the lesion, as well as the significance of clinical data for making an accurate diagnosis. The importance of clinical data is further supported by the superior performance of the clinical–radiomics model compared with the radiomics model. It is likely that color Doppler ultrasound information, which was available only to the original ultrasound examiners, also contributes substantially to a correct diagnosis of sarcoma *vs* leiomyoma^12^.

A model incorporating both clinical and radiomics variables has the potential to improve management of uterine tumors. Ultrasound machines with validated radiomics‐based models and automated radiomics analysis embedded in their software could improve the discrimination between uterine sarcomas and leiomyomas. However, radiomics analysis is currently largely a research tool, with several hurdles to be overcome before its clinical implementation. These include, for example, the computational demands of image processing and feature extraction, the need to standardize workflows (e.g. regarding whether to acquire DICOM or joint photographic experts group (JPEG) images, to analyze two‐dimensional images or three‐dimensional ultrasound volumes, how many images per lesion to analyze) and the need to ensure interoperability across different ultrasound platforms. Additionally, regulatory requirements must be addressed, as radiomics software would need to be approved as a medical device^46^. Despite these challenges, with continued technological advances, the integration of radiomics into routine clinical practice may become feasible in the near future.

To estimate the ability of a radiomics model to discriminate between all types of benign and malignant uterine smooth muscle tumors, a very large prospective study, including all types of smooth muscle tumors, as well as benign variants of uterine leiomyomas and STUMP, is needed. Such a study should include a comparison of the radiomics model's performance with that of subjective assessment of ultrasound images reviewed by expert ultrasound examiners and with that of the individuals performing the original ultrasound examination.

## Supporting information


**Table S1** Participating centers and number of tumors contributed per center.
**Tables S2 and S3** Model performance for all machine‐learning classifiers (logistic regression, random forest, extreme gradient boosting (XGBoost), support vector machine (SVM)) used for building radiomics model (Table S2) and clinical–radiomics model (Table S3), calculated for best cut‐off based on Youden's index.
**Table S4** Discriminative and classification performance of delta‐radiomics model.
**Appendix S1** Details of the selected radiomics features.
**Figures S1 and S2** Receiver‐operating‐characteristics (ROC) curves for discrimination between uterine sarcomas and leiomyomas using radiomics model (Figure S1) and clinical–radiomics model (Figure S2), across different classifiers (logistic regression, random forest, extreme gradient boosting (XGBoost), support vector machine (SVM)) for: (a) training (*n* = 140) and (b) validation (*n* = 60) sets. Best cut‐offs according to Youden's index are indicated (circles).
**Figure S3** Boxplots showing distribution of the delta‐radiomics features used for radiomics modeling, stratified by tumor group (leiomyomas and sarcomas).

## Data Availability

The data that support the findings of this study are available from the corresponding author upon reasonable request.
