# Supplementary material for: Radiomics‐based ultrasOund Model for differentiating Uterine Sarcomas from leiomyomas (ROMUS): a retrospective pilot Multicenter Italian Trials in Ovarian Cancer (MITO) study
Source: Ultrasound Obstet Gynecol. 2026 Mar 6;67(4):530–41. doi: 10.1002/uog.70187 (PMC13040125; doi:10.1002/uog.70187)

**SUPPORTING INFORMATION**

| **Table S1** Participating centers, showing number of tumors contributed per center. | | | | | |
| --- | --- | --- | --- | --- | --- |
| Characteristics | Oncological Center | Hospital  (Regional/  Local/university) | All cases | Leiomyomas | Sarcomas |
|  |  |  | n=200 | n=100 | n=100 |
| Fondazione Policlinico Universitario A. Gemelli - IRCCS - Roma, Italy | Yes | University | 106 (53.0) | 61 (61.0) | 45 (45.0) |
| I.R.C.C.S. Burlo Garofalo - Trieste, Italy | Yes | Regional | 24 (12.0) | 12 (12.0) | 12 (12.0) |
| Skane University Hospital, Lund University of Malmo - Malmo, Sweden | No | University | 22 (11.0) | 11 (11.0) | 11 (11.0) |
| Ospedale di Varese -Varese, Italy | Yes | Regional | 16 (8.0) | 8 (8.0) | 8 (8.0) |
| Città della Salute e della Scienza Ospedale S. Anna - Torino, Italy | Yes | University | 7 (3.5) | 0 (0.0) | 7 (7.0) |
| IRCCS Istituto Tumori di Bari Giovanni Paolo II - Bari, Italy | Yes | Regional | 5 (2.5) | 0 (0.0) | 5 (5.0) |
| IRCCS Policlinico S. Matteo - Pavia, Italy | Yes | University | 4 (2.0) | 2 (2.0) | 2 (2.0) |
| Ospedale Giovan Battista Morgagni - Forlì, Italy | Yes | University | 4 (2.0) | 2 (2.0) | 2 (2.0) |
| Ospedale San Raffaele - Milano, Italy | Yes | University | 4 (2.0) | 2 (2.0) | 2 (2.0) |
| Ospedali Infermi e Presidio di Oncologia - Rimini Cattolica, Italy | No | University | 2 (1.0) | 0 (0.0) | 2 (2.0) |
| Ospedale Regionale Miulli - Acquaviva delle Fonti, Italy | Yes | Regional | 2 (1.0) | 1 (1.0) | 1 (1.0) |
| Ospedale S.Orsola Università di Bologna - Bologna, Italy | Yes | University | 2 (1.0) | 0 (0.0) | 2 (2.0) |
| I.N.T.di Roma, Italy Regina Elena - Roma, Italy | Yes | Regional | 2 (1.0) | 1 (1.0) | 1 (1.0) |
| Results are presented as n (%). |  |  |  |  |  |

**Table S2** Model performances for all machine-learning classifiers (logistic regression, random forest, XGBoost, support vector machine (SVM)) used for building radiomics model, calculated for best cut-off based on Youden´s index (obtained in training set then applied in validation set).

|  | LOGISTIC REGRESSION  Youden’s cut-off: 46 % | | RANDOM FOREST  Youden’s cut-off: 53 % | | XGBOOST  Youden’s cut-off: 47 % | | SVM  Youden’s cut-off: 49 % | |
| --- | --- | --- | --- | --- | --- | --- | --- | --- |
|  | Train | Valid | Train | Valid | Train | Valid | Train | Valid |
| AUC | 0.85  (0.77- 0.91) | 0.86  (0.75 -0.94) | 0.87  (0.81- 0.92) | 0.89  (0.79-0.97) | 0.93  (0.88-0.96) | 0.87  (0.76 -0.96) | 0.84  (0.77 - 0.90) | 0.86  (0.76 - 0.94) |
| Accuracy | 0.80  (0.73 -0.87) | 0.80  (0.70 - 0.90) | 0.80  (0.73 - 0.87) | 0.80  (0.70 - 0.90) | 0.86  **(**0.81- 0.92) | 0.85  (0.76 - 0.94) | 0.79  (0.72 - 0.85) | 0.85  (0.72 - 0.92) |
| Sensitivity | 0.84  (0.76 -0.93) | 0.83  (0.70 - 0.97) | 0.73  (0.62 - 0.83) | 0.73  (0.58 - 0.89) | 0.89  (0.81 - 0.96) | 0.87  (0.74 - 0.99) | 0.84  (0.76 - 0.93) | 0.87  **(**0.74 - 0.99) |
| Specificity | 0.76  (0.66 -0.86) | 0.77  (0.62 - 0.92) | 0.87  (0.79 - 0.95) | 0.87  (0.74 - 0.99) | 0.84  (0.76 - 0.93) | 0.83  (0.70 - 0.97) | 0.73  (0.62 - 0.83) | 0.77  (0.62 - 0.92) |

Values in brackets are 95% confidence intervals (CIs) constructed using bootstrap for area under the receiver-operating-characteristics curve (AUC) and normal approximation for all other measures.

**Table S3** Model performances for all machine-learning classifiers (logistic regression, random forest, XGBoost, support vector machine (SVM)) used for building clinical–radiomics model, calculated for best cut-off based on Youden’s index (obtained in training set and applied in validation set).

|  | LOGISTIC REGRESSION  Youden’s cut-off: 52 % | | RANDOM FOREST  Youden’s cut-off: 47 % | | XGBOOST  Youden’s cut-off: 47 % | | SVM  Youden’s cut-off: 45 % | |
| --- | --- | --- | --- | --- | --- | --- | --- | --- |
|  | Train | Valid | Train | Valid | Train | Valid | Train | Valid |
| AUC | 0.87  (0.81 - 0.93) | 0.90  (0.81 - 0.97) | 0.98  (0.97 - 0.997) | 0.93  (0.86 - 0.99) | 0.98  (0.97 - 1) | 0.93  (0.86 - 0.99) | 0.91  (0.86 - 0.96) | 0.90  (0.81 - 0.97) |
| Accuracy | 0.81  (0.74 - 0.87) | 0.85  (0.76 - 0.94) | 0.94  (0.90 - 0.98) | 0.85  (0.76 - 0.94) | 0.94  (0.90 - 0.98) | 0.88  (0.70 - 0.97) | 0.85  (0.79 - 0.91) | 0.85  (0.76 - 0.94) |
| Sensitivity | 0.87  (0.79 - 0.95) | 0.90  (0.79 - 1) | 0.91  (0.85 - 0.98) | 0.87  (0.74 - 0.99) | 0.90  (0.83 - 0.97) | 0.93  (0.84 - 1) | 0.89  (0.81 - 0.96) | 0.87  (0.75 - 0.99) |
| Specificity | 0.74  (0.64 -0.85) | 0.80  (0.66 - 0.94) | 0.96  (0.91, 1) | 0.83  (0.70, 0.97) | 0.97  (0.93 - 1) | 0.83  (0.70 - 0.97) | 0.81  (0.72 - 0.91) | 0.83  (0.70 - 0.97) |

Values in brackets are 95% confidence intervals (CIs) constructed using bootstrap for area under the receiver-operating-characteristics curve (AUC) and normal approximation for all other measures.

**Table S4** Discriminative and classification performance of delta-radiomics model.

| AUC​ | | Accuracy​ | Sensitivity​ | Specificity​ |  |
| --- | --- | --- | --- | --- | --- |
| Model fitting  Cross-Validation | | 0.81 (0.69-0.91)  0.78 (0.07) | 0.78 (0.68-0.88)  0.73 (0.09) | 0.78 (0.64-0.92)  0.71 (0.01) | 0.78 (0.64-0.92)  0.74 (0.08) |

Accuracy, sensitivity and specificity are calculated for the 49 % risk of malignancy cutoff, i.e. the best cutoff based on Youden´s index calculated during model fitting. The 95% confidence intervals (CI) are reported for model fitting metrics in parentheses with bootstrapping being used to estimate the CI of the AUC and normal approximation to estimate the CI of the classification metrics. Mean and standard deviation (in parentheses) are reported for the cross-validation. AUC, area under the receiver operating characteristics curve.

**Appendix S1** Details of the selected radiomics features.

The selected radiomics features are categorized into the following feature families: intensity-based statistical features (F_stat), textural features derived from the gray-level co-occurrence matrix (F_cm), textural features derived from the gray-level run-length matrix (F_rlm), and textural features derived from the gray-level size zone matrix (F_szm).
The full names of the selected radiomics features can be found in the Image Biomarker Standardization Initiative (IBSI) reference manual.

- Statistical features

| F_stat.rms | Root mean square |
| --- | --- |

- Textural features based on the gray-level co-occurrence matrix

| F_cm.joint.entr | Joint entropy |
| --- | --- |
| F_cm.clust.shade | Cluster shade |
| F_cm.inv.var | Inverse variance |
| F_cm.info.corr.2 | Second measure of information theoretic correlation |

- Textural features based on the gray-level run length matrix

| F_rlm.lrhge | Long run high gray level emphasis |
| --- | --- |

- Textural features based on the gray-level size zone matrix

| F_szm.lze | Large zone emphasis |
| --- | --- |
| F_szm.glnu | Gray level non-uniformity |

**Figure S1** Receiver-operating-characteristics (ROC) curves for radiomics model of different classifiers (logistic regression, random forest, XGBoost, support vector machine (SVM)) for: (a) training (*n*= 140) and (b) validation (*n*= 60) sets. Best cut-offs according to Youden’s index are indicated (circles).

A B


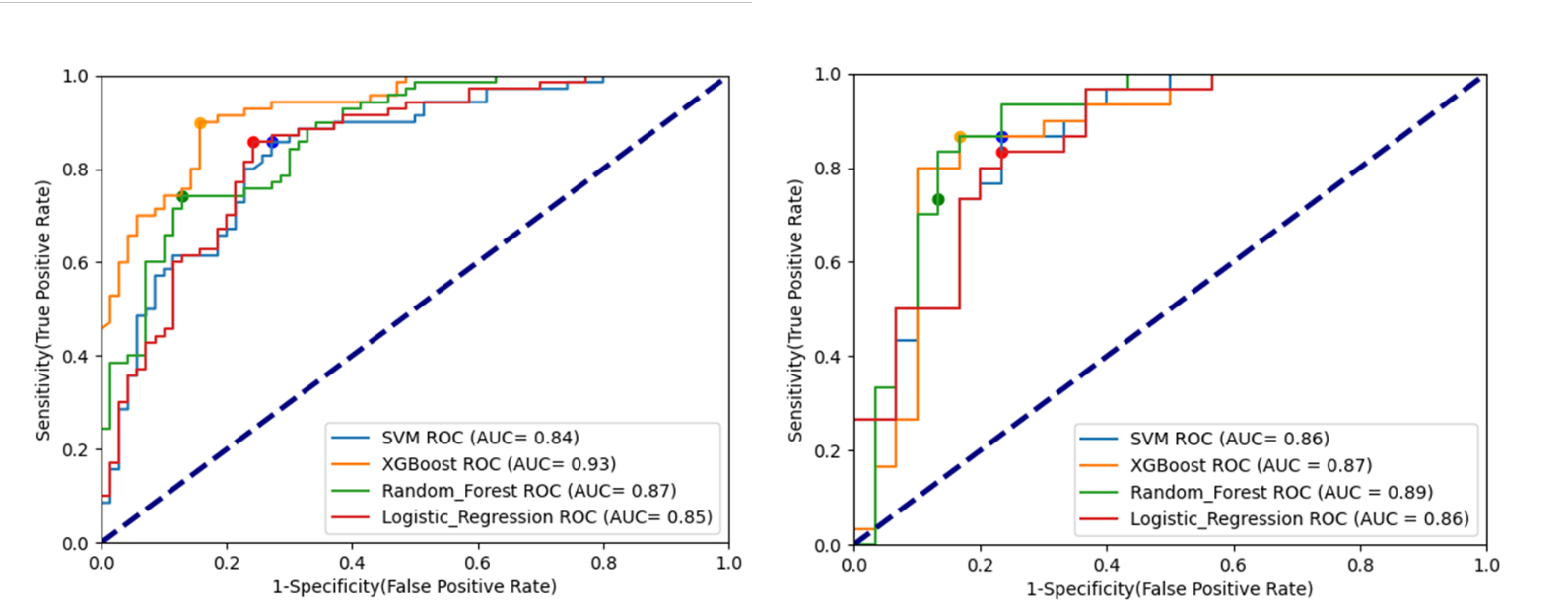


**Figure S2** Receiver-operating-characteristics (ROC) curves for clinical–radiomics model of different classifiers (logistic regression, random forest, XGBoost, support vector machine (SVM)) for: (a) training (*n*= 140) and (b) validation (*n*= 60) sets. Best cut-offs according to Youden’s index are indicated (circles).

A B


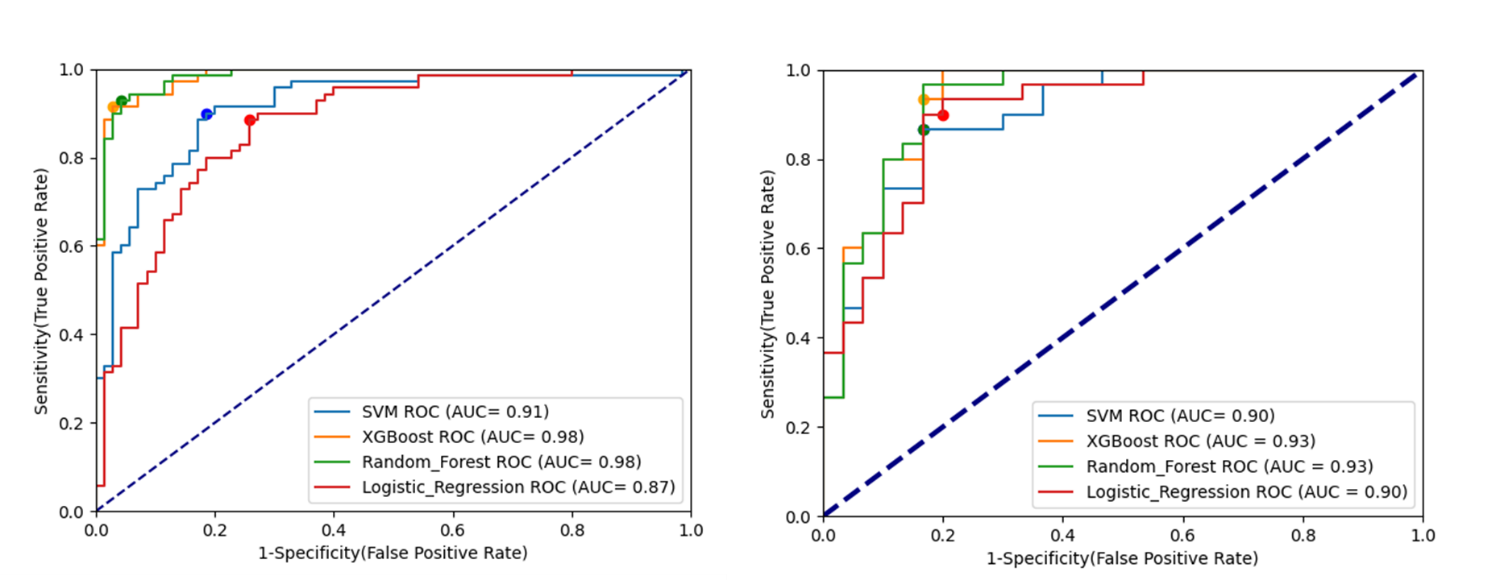


**Figure S3** Box plot distribution of the delta-radiomics features used for radiomics modeling stratified by tumor group (leiomyomas and sarcomas). Boxes represent interquartile range (IQR), which contains the middle 50% of the values. The line inside the box indicates the median. Whiskers correspond to values within 1.5 times of the IQR. Dots are outliers. The *P*-values show results of the Wilcoxon–Mann–Whitney test.


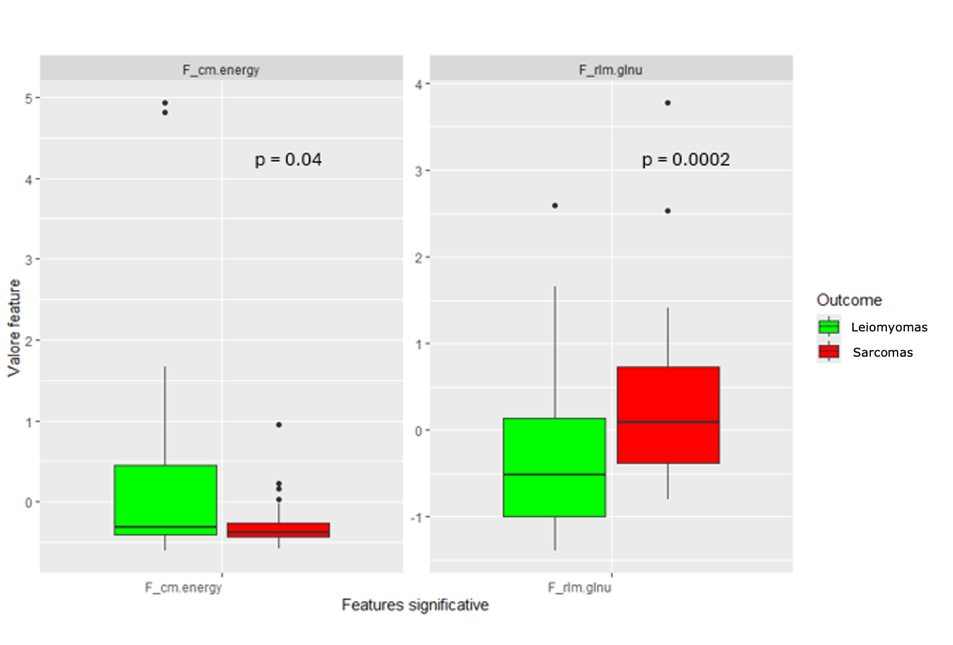

Supplement: Supplementary file 1 — Table S1 Participating centers and number of tumors contributed per center. Tables S2 and S3 Model performance for all machine‐learning classifiers (logistic regression, random forest, extreme gradient boosting (XGBoost), support vector machine (SVM)) used for building radiomics model (Table S2) and clinical–radiomics model (Table S3), calculated for best cut‐off based on Youden's index. Table S4 Discriminative and classification performance of delta‐radiomics model. Appendix S1 Details of the selected radiomics features. Figures S1 and S2 Receiver‐operating‐characteristics (ROC) curves for discrimination between uterine sarcomas and leiomyomas using radiomics model (Figure S1) and clinical–radiomics model (Figure S2), across different classifiers (logistic regression, random forest, extreme gradient boosting (XGBoost), support vector machine (SVM)) for: (a) training (n = 140) and (b) validation (n = 60) sets. Best cut‐offs according to Youden's index are indicated (circles). Figure S3 Boxplots showing distribution of the delta‐radiomics features used for radiomics modeling, stratified by tumor group (leiomyomas and sarcomas). [file UOG-67-530-s001.docx]
